# Are All Data Created Equal? - Exploring Some Boundary Conditions for a Lazy Intuitive Statistician

**DOI:** 10.1371/journal.pone.0097686

**Published:** 2014-05-16

**Authors:** Marcus Lindskog, Anders Winman

**Affiliations:** Department of Psychology, Uppsala University, Uppsala, Sweden; International School for Advanced Studies, Italy

## Abstract

The study investigated potential effects of the presentation order of numeric information on retrospective subjective judgments of descriptive statistics of this information. The studies were theoretically motivated by the assumption in the naïve sampling model of independence between temporal encoding order of data in long-term memory and retrieval probability (i.e. as implied by a ”random sampling” from memory metaphor). In Experiment 1, participants experienced Arabic numbers that varied in distribution shape/variability between the first and the second half of the information sequence. Results showed no effects of order on judgments of mean, variability or distribution shape. To strengthen the interpretation of these results, Experiment 2 used a repeated judgment procedure, with an initial judgment occurring prior to the change in distribution shape of the information half-way through data presentation. The results of Experiment 2 were in line with those from Experiment 1, and in addition showed that the act of making explicit judgments did not impair accuracy of later judgments, as would be suggested by an anchoring and insufficient adjustment strategy. Overall, the results indicated that participants were very responsive to the properties of the data while at the same time being more or less immune to order effects. The results were interpreted as being in line with the naïve sampling models in which values are stored as exemplars and sampled randomly from long-term memory.

## Introduction

People often make intuitive statistical judgments from previously experienced data with little or no information about the upcoming judgment before any data is presented. A recent framework for intuitive statistical judgments has suggested that people approach such judgments as *naïve intuitive statisticians* [1]–[5] and that the generic cognitive process they engage in could be described by the *naïve sampling model* (NSM) [4]. The NSM suggests that judgments of statistical properties are computed on small samples of observations retrieved form memory at the time of judgment [1], [4], a strategy that resembles *lazy algorithms* [1], [5] (In making the distinction between lazy and eager algorithms throughout this paper, we intend to make a qualitative comparison on a larger scope between these concepts. Thus, the aim is not to test the performance of any particular implemented algorithm quantitatively.) that can be found both in cognitive science [6] and machine learning [7]. While a lazy strategy affords computational flexibility in complex situations it requires, for efficiency, that a sufficiently large portion of undistorted data is accessible from memory at the time of a judgment. As of yet, little research has addressed if this is the case for intuitive statistical judgments. In addition, a lazy strategy requires a minimum of assumptions about the experienced variable in order to represent statistical properties. However, few studies have addressed the extent to which people enter laboratory tasks with assumptions about properties of the data.

In the following, we first review research concerned with people's ability to act as intuitive statisticians for a variety of statistical properties. The second part of the introduction reviews evidence suggesting that people use lazy strategies to form intuitive statistical judgments and research investigating order effects in judgments of statistical properties. We then present two experiments investigating *a*) if people sample from all of the experienced data at the time of a query in a random fashion, or if memory effects, similar to *primacy* and *recency*, influence intuitive statistical judgments and, *b*) if people enter our laboratory task with expectations about the properties of data. Here we take *primacy* and *recency* to mean effects on memory that could be accounted for by the data being presented either early on or late in a sequence. Thus, primacy and recency is not used in the perhaps more strict sense found in research investigating the serial position effect.

### Intuitive Judgments of Statistical Properties

To what extent are people able to estimate the statistical properties of experienced data? The research investigating this question has mainly focused on the extent to which estimates of descriptive properties (e.g., central tendency and variability) are normative. This research has found that while some properties (e.g., central tendency) are often accurately reported, others (e.g., variability) are consistently biased [8], [9]. Even though early research in the field depicted people as able *intuitive statisticians* [10] later research has indicated that statistical judgments are often informed by inefficient heuristics and subject to biases [11], [12].

The focus of much of the previous research has been on lower level properties (first and second moment) of the experienced data. However, using primarily variables encountered in everyday life, some studies [13]–[17] have also investigated people's ability to estimate higher order properties, like distribution shape. The study of higher order properties, although to a lesser extent, has also been conducted in laboratory settings with variables being experienced by participants on a trial-by-trial basis [1], [5], [18]. The results from studies on higher order properties, with respect to accuracy, are mixed. Some studies report very accurate judgments [15], [18] whereas other studies indicate that factors such as what information is accessible in the environment [14], [16], where people find themselves in the distribution [3], [17], and the properties of the underlying distribution [1], [14] influence the accuracy of judgments. In addition, researchers have investigated if people can use their knowledge of higher order properties to make predictions [5], [15], [18]. Some of these studies indicate that people can use their knowledge to make remarkably accurate predictions [15], [18] while others suggest that accuracy is dependent on properties of the underlying variable [5].

### Lazy or Eager: The Cognitive Process of Statistical Judgments

With a few exceptions [1], [5], [19], and in contrast to related areas like categorization learning [20], [21], multiple-cue judgments [22], [23], and function learning [24], [25], research concerned with statistical judgment has paid little attention to the cognitive process underlying the judgment. However, a recent framework for statistical judgments [2]–[4], where people are considered naïve intuitive statisticians, has suggested a general process model for how some statistical judgments are formed.

The general model is derived from the NSM [4], originally outlined for intuitive confidence intervals, that describes how people realize their knowledge of an experienced variable. The model describes how data is handled during encoding and when making a judgment. According to the model, all experienced data is stored in long-memory (LTM) as raw data points (exemplars) during exposure and no operations are carried out on the data. When a person is queried for a judgment, a small random sample of experienced data is retrieved from memory and becomes active in short-term memory (STM). Finally, calculations of statistical properties are carried out on the small sample active in STM and the results are used by the person as a proxy for population properties [1], [4], [5]. The model thus describes a *lazy* cognitive algorithm operating on experienced data to make intuitive judgments. The features of the process imply that judgments will be constrained by several cognitive limitations. First, the size of the samples on which computations are made will have to be of a size that can be activated in STM, often estimated to 4±2 [26]. Second, the information integration will be constrained by the sequential real-time properties of a controlled judgment process [4]. Further, because some sample properties (e.g., mean and proportion) are unbiased under random sampling while others (e.g., variance and coverage) are not, the resulting judgments will tend to be accurate for the former but not for the latter type of property.

The lazy process model outlined above has been contrasted against an *eager* process model for statistical judgments [1], [5]. An eager model assumes that summary information is extracted from the data on-line during exposure to the variable. The process thus resembles the spontaneous calculation of the intuitive equivalents of a running mean or variance, updated as each new data point is being presented.

The distinction between an eager and a lazy cognitive model is similar to the distinction between “eager” and “lazy” learning algorithms in artificial intelligence [7] and that between on-line and retrospective models in cognitive science [27]. Lazy algorithms, rather than pre-computing statistical summaries for every conceivable future demand, postpone computations until the specific need of them is specified by a query. While the pre-computed summary statistics of eager algorithms may succeed well in closed and well defined environments, it has been suggested [6], [27] that lazy algorithms, such as exemplar models [21], the NSM [1], [4], [5], the Minerva 2 model [28], and the enumeration model [29], afford greater efficiency and flexibility. It has even been suggested that models relying on on-line computations of statistical properties (e.g., cue validities) quickly become computationally intractable as the complexity of the environment increases [27]. In addition, previous research has shown that people at least under specific circumstances may have access to data after encoding [30], in contrast to what is expected by the destructive nature of models that extract descriptive parameters during exposure and then disregard the data, and that estimates of descriptive parameters seem to be constrained by STM capacity [31]. Further, statistical judgments such as confidence intervals [4], point predictions [5], and proportions [1] seem to be generated by a lazy cognitive algorithm. In sum: both theoretical arguments and previous empirical findings indicate support for the idea that people, in general, form statistical judgments by computations made on small samples drawn post hoc from memory.

Several research areas assume that people have access to or can generate estimates of statistical properties, without necessarily specifying the process by which this is done [32]. In Bayesian accounts of human cognition [33], [34], for example, cognitive processes are thought to be adaptations to distributions in the environment and it is reasonable to assume that these environmental distributions need to be represented in memory and made available at the time of a judgment. Further, research concerned with binary gambles has seen a recent interest in tasks where probabilities and outcomes are learned experientially [35], rather than by explicit top down verbal information [36], thereby requiring participants to generate statistical judgments from memory at the time of a query. Also, recent work on social judgments has suggested that people infer how other people are doing (e.g., the distribution of income) by sampling data available in their immediate social environment [14]. Because the use of one cognitive algorithm over another is likely to enforce boundary conditions whenever a statistical judgment is made, like in the examples above, the question of whether statistical judgments are generated from a lazy or an eager process is thus not exclusively of interest to the specific area of statistical judgments.

While there is reasonable evidence to support the claim that people generally construct statistical judgments with a lazy cognitive algorithm, there are several boundary conditions and assumptions for a possible lazy process that are yet to be empirically explored. First, because the lazy model includes raw data being stored and retrieved from LTM, common memory effects such as primacy and recency may influence judgments. Indeed, some previous research has indicated that this might be the case [31]. In the extant formulation of the NSM all data are treated equal in sampling, and by this account it thus predicts the absence of either primacy or recency effects. However, it may well be the case that this assumption, that human judges have the ability to override the common-day memory phenomena of primacy/recency, is overly optimistic.

Second, a defining feature of eager cognitive algorithms is that they require assumptions about the experienced variable (e.g., the distribution shape) in order to store higher order properties [1]. In contrast, lazy algorithms require a minimum of such assumptions. Even though previous research has indicated that people enter some statistical tasks with strong prior assumptions [37], [38] it is still an empirical question if similar assumptions exists for higher order statistical properties.

### Order Effects in Judgments of Statistical Properties

Studies examining effects of presentation order on statistical judgments are almost entirely missing when it comes to descriptive statistics of univariate numeric information. In contrast, a number of studies have examined such effects on judgments of contingency or covariation between two variables. The reason for the interest in order effects in this area is the aim to distinguish between associative and rule based theories [39] of how judgments of this statistical property are formed. According to rule based accounts, people encode specific exemplars of each piece of information and calculate the contingency from these exemplars by applying a specific rule [39]. According to the associative account, contingency judgments are based on accumulating changes in associative strength updated on a trial-by-trial basis by connectionist learning rules. The emphasis in rule-based accounts is thus on higher order cognitive reasoning like processes, whereas associative accounts concern lower level conditioning-like processes present in animals and infants. If judgments are made on basis of all exemplars stored in memory, as typically in rule based accounts the order in which the information is presented should have no effect. On the other account, however, such as connectionist implementations of the Rescorla-Wagner model [40], fundamental order effects are predicted, in which the most recent information will have the largest impact on judgments [41]. Whereas some studies [42] have obtained such recency effects, others [43], have instead found a primacy effect inconsistent with the Rescorla-Wagner model. Glautier [45] found a primacy effect, but showed that this effect could be reversed by requesting judgments by participants in the midst of the information presentation sequence.

To summarize: There is a shortage of studies on order effects on descriptive statistics of numeric information. Several studies of contingency judgments have shown profound order effects at odds with both rule based and associative accounts, and found strong effects of whether or not judgments are requested intermixed with information presentation. The aim of the present study is to examine if corresponding order effects exist for judgments of descriptive statistics of numeric information presented sequentially, or whether these are consistent with judgments of an intuitive statistician who stores, and has access to, all encountered data.

### The Present Study

In the present study we conducted two experiments aimed at addressing two major questions. First, to what extent do people have access to all data encountered during exposure in subsequent subjective statistical judgments? Note that by this we do not imply that all of the data is incorporated in the final judgment but rather that all of the data is equally available for sampling by the cognitive algorithm that produces the judgment, irrespective of presentation order. Put differently, having access to all the data would be equivalent to randomly sampling from all of the encountered data at the time of a query while possible memory effects would imply sampling conditionally on whether the data was encountered early on (primacy) or late (recency) during encoding. Second, do people enter our laboratory task with expectations about the statistical properties of the data?

In both experiments participants experienced a numerical variable on a trial-by-trial basis. They were later asked to perform a series of tasks designed to evaluate their knowledge of the statistical properties of the experienced variable. In Experiment 1 we investigated whether memory effects such as primacy and/or recency are present for statistical judgments. We designed Experiment 2 to rule out alternative interpretations of the results of the first experiment. In addition, the design of Experiment 2 allowed us to evaluate the extent to which participants enter the laboratory task with expectations about the properties of the data and whether people have the ability to update their previously expressed beliefs when the structure of the information suddenly changes, or if they do anchor on these prior beliefs.

Further, both an eager and a lazy cognitive algorithm presuppose that participants inform statistical judgments by a computation of properties on experienced data. It is, however, possible that some tasks could be solved successfully without such computations if memory for specific values could be used directly. To address this possibility, we also included a manipulation, in both experiments, designed to evaluate whether the observed responses were due to memory for specific values or if participants made inferences of statistical properties from the data.

## Experiment 1

**Figure 1:**
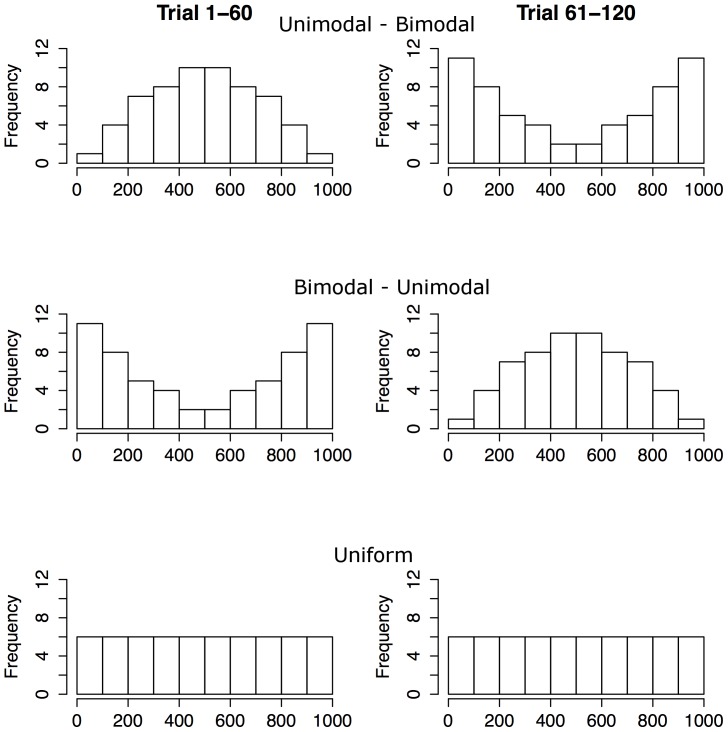
Illustration of the design of the learning phase of Experiment 1 and 2.

**Figure 2:**
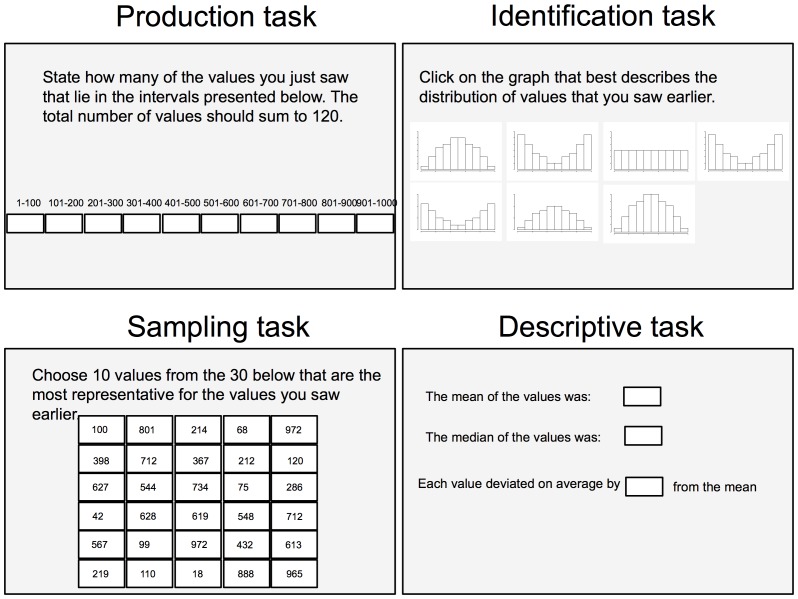
Illustration of the tasks used in the test phase of Experiment 1 and 2.

During learning participants observed 120 uniformly distributed values. The values were presented in one of three sequential orders (described below) with markedly different distributions for the first and last 60 values. The design is illustrated in Figure 1. Participants were later asked to perform a set of tasks designed to measure their knowledge of the statistical properties of these numbers. These tasks, described below, are illustrated in Figure 2. The experiment was designed to investigate if memory mechanisms influence statistical judgments.

### Method

#### Ethics Statement

In both experiments of the present study all participants received an information sheet on the study and provided verbal informed consent before undertaking the study. The nature of the study was not in any way invasive, or unpleasant, did not involve deception, part taking was voluntary and participants were explicitly told that they could abort the study whenever they wished. In addition, no personal information was recorded. There was no further documentation of the informed consent, and such documentation was not a requirement of the ethic committee. The ethic committee of Uppsala University approved the research and the consent procedure. In accordance with the recommendations of the American Psychological Association, the data from the study are available on request.

#### Participants

Participants were 48 undergraduate (12 male) students from Uppsala University (*M* = 24.5 years, *SD* = 4.8) receiving a movie voucher or course credit for participating.

#### Materials and procedure

The computerized task consisted of an exposure phase and a test phase. During exposure, participants observed values described as “test player ratings of a fictitious computer game”. They were told that the ratings were a random sample of ratings from all test players and that the ratings were in a 1 to 1000 range. Participants' task was to “observe the ratings carefully in order to answer questions about them at a later point”.

Two sets (*E* and *T*) of 120 numbers were uniformly sampled from the interval [1, 1000] with the constraint that each of the subintervals [1, 100], [101, 200] … [901, 1000] contained 12 values. One of the sets, *E*, was presented during exposure while the other, *T*, was withheld until the sampling task (see below) of the test phase. Each of the values in *E* was shown once during exposure accompanied by a nonsense player identification code. Presentation was self-paced but each number remained on the screen for a minimum of 3 seconds before participants could proceed. The same sets of values were used for all participants.

A partitioning of *E* into two subsets (*E_a_* and *E_b_*) of 60 values defined the three conditions. In the *unimodal-bimodal* (U-B) and *bimodal-unimodal* (B-U) conditions the values of *E_a_* were chosen to be unimodally distributed (Beta distribution with shape parameters [2.4, 2.4]). The remaining values, *E_b_*, were then bimodally distributed, where *E_b_* is the reflection of *E_a_* with respect to a uniform distribution. Participants in the U-B-condition experienced the *E_a_* set first followed by the *E_b_* set while the order was reversed in the B-U-condition. In the *uniform* (UN) condition the values for *E_a_* and *E_b_* were distributed randomly across order positions 1–120, with a resulting uniform distribution of values. All values thus only occurred once. All participants were given an independent presentation order given the above constraints. The transition from *E_a_* to *E_b_* was not announced or in any other way implied in any condition. Participants were randomly assigned to one of the three conditions with an equal number of participants (*n* = 16) in each condition. The test phase included four tasks described below.

In the *identification task*, participants chose one of 7 histograms that ”best described the distribution of the numbers”. One graph was uniform, three were unimodal with decreasing variance and three were bimodal with increasing variance. An explanation of the graphs was given prior to their presentation. The explanation included explicit exemplifications (e.g., ”A graph which is higher to the sides than in the middle indicates that most of the values were either high or low.”). The graphs were provided without metric information on the axes.

In the *production task*, **p**articipants assessed how many of the values from *E* that fell into ten equally wide intervals ([1, 100], [101, 200] … [901, 1000]) with frequencies required to sum to 120.

Each of the 8 trials in the *sampling task* presented participants with a 5×6 matrix containing 30 values. Matrices were presented one at a time and participants were asked to choose 10 values out of the 30 presented values in each matrix. They were asked to choose the 10 values from each matrix that “were the most representative of the experienced values”. Four of the matrices contained values from *E* (old) and four contained values from *T* (new). The values in each matrix were distributed uniformly in [1, 1000]. Prior to the task participants were given explicit details of how to interpret the instructions. It was explained that the sample being representative of *E* meant that it should not be systematically different from *E* (i.e., to have the same properties as *E*) and that this could occur without any specific values from *E* being present in the sample.

In the *descriptive task*, participants estimated the central tendency (mean and median) and variability (mean absolute deviation; MAD) of *E*. They were given a brief definition of the measures in terms of an explicit exemplification of its calculation (e.g. “The mean for a set of numbers is the sum of the numbers divided by the count of the numbers. For example, the mean of 4, 8, 12 is 8 because (4+8+12)/3 = 8.”, “The median is the middle value in a list of all values arranged from the lowest to the highest value.” Mean absolute deviation was explained as “The mean of the distances of each value from their total mean.”). The order of the three first tasks was counterbalanced over participants while the descriptive task was carried out last.

### Results

#### Central tendency and variability

**Figure 3:**
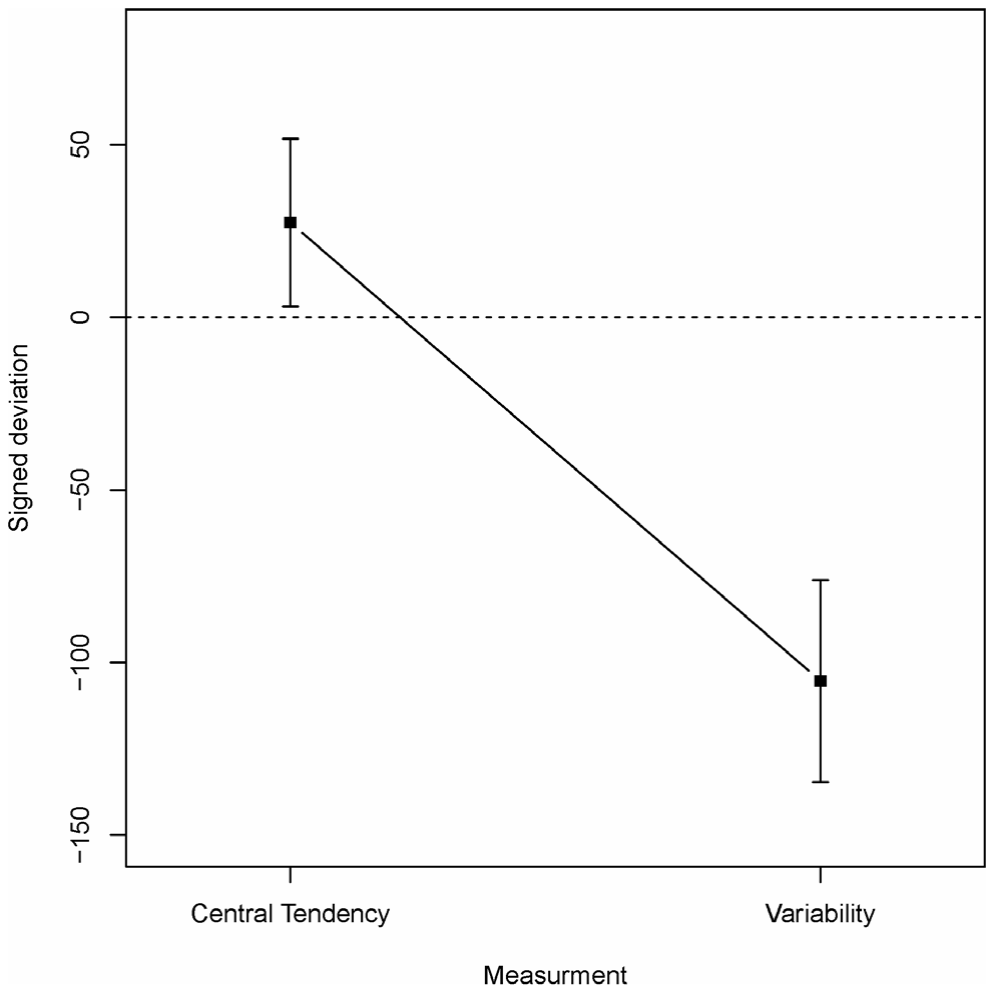
Signed deviation of estimates of Central tendency and Variability in Experiment 1. Dashed line indicates unbiased estimates. Vertical bars denote 95%-confidence intervals.

Participants gave estimates of both mean and median. There was no difference in the accuracy of these estimates and the data were therefore collapsed to create one measure of central tendency. To investigate both accuracy and possible bias of estimates of central tendency and variability we calculated absolute and signed deviation, respectively, between participants' estimates and the normative value of each statistic. One outlier was removed from the analyses reported below. The absolute and signed deviations were calculated as 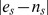 and 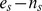 respectively, where *e_s_* is the participant's estimate of the statistic and *n_s_* is the normative value of the statistic. The absolute deviation was entered as dependent variable into a 3×2 mixed ANOVA with condition (U-B/B-U/UN) as between-subjects independent variable and measurement type (central tendency/variability) as within-subjects independent variable. For all ANOVAs reported below, Levene's test of homoscedasticity was performed. In no case did the test indicate violation of this assumption. The analysis revealed a significant main effect of measurement type (*F*(2, 44) = 10.9, *p* = .002) with better estimates (for the absolute deviation) of central tendency (*M* = 78.6, *SD* = 46.7) than of variability (*M* = 119.6, *SD* = 81.7). Neither the effect of condition nor the interaction effect was significant (*F*s<1). A corresponding ANOVA for the signed difference investigated a possible bias in estimates. The significant main effect of measurement type (*F*(2, 44) = 50.2, *p*<.001) is illustrated in Figure 3, which shows that central tendency is slightly overestimated while variability is underestimated to a large extent. Neither the effect of condition (B-U: *M* = −42.4, *SD* = 117.6; U-B: *M* = −60.1, *SD*  = 110.5; UN: *M* = −10.6, *SD* = 108.0) nor the interaction reached significance (both *p*s >.11). With the current design, with symmetric distributions, the average mean presented to participants is necessarily constant in the first and second half of the data. However, since participants received individual random sequences of numbers it is possible to check for order effects on mean estimates by examining ratings compared to the means actually occurring early on/late in the sequence. We performed this analysis on the first/last quarter (30 trials) for each participant. Because we were primarily interested in the impact of data presented early on/late in the sequence we did not include the intermediate data in the analysis. The absolute error based on the first/last/total mean experienced by participants was entered into a 3×3 mixed ANOVA with condition as between-subjects variable and deviation from first/last/total mean as within-subjects variable. There were no significant effects, and errors were slightly larger for the first/last quarter than for the total mean, thus suggesting neither primacy nor recency effects. This partitioning of stimuli into first/second quarter is in some sense arbitrary. We therefore performed corresponding analyses for various other partitions with no signs of primacy or recency effects.

#### Performance measure for knowledge of distribution shape

To evaluate participants' knowledge of the distribution shape of *E* we calculated a shape sensitive index (*Shape Index*; SI) for each of the three tasks. With the range [1, 1000] divided in to 10 intervals [1, 100], [101, 200] … [901, 1000] numbered according to 1…*i*…10, SI is given by
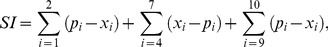
(1)where *x_i_* is the participant's judgment of the proportion of the distribution in the *i*:th interval and *p_i_* is the normative proportion that had been observed by participants (i.e., because the underlying distribution is uniform, values within each of ten intervals occur with proportion .1). The 301–400 and 801–900 intervals are excluded in the formula because these intervals are uninformative with respect to the unimodal/bimodal distinction of interest. In the production task *x_i_* is the frequency given explicitly by the participant divided by 120. In the identification task *x_i_* was given by the corresponding values calculated for the chosen graph (because each graph depicted a distribution through a histogram). Finally, in the sample task, values chosen by the participant were categorized into the ten intervals and the proportion of values in each interval gave *x_i_*. Thus, for all tasks SI is both a measure of the degree to which estimates deviate from the underlying uniform distribution and sensitive to the shape of the estimated distribution. SI = 0 will indicate that judgments are uniform while SI>0 and SI<0 will indicate an estimated “subjective” distribution that is unimodal and bimodal respectively. SI was defined in line with our goal to investigate systematic biases towards unimodality/bimodality. This measure cannot capture strongly skewed distributions. In analyzing data we have not found such tendencies towards skewed distributions. We also performed calculations on a mean absolute error defined as the corresponding unsystematic deviations. Since this measure did not add much new information we have refrained from presenting these analyses not to overburden the exposition.

#### Estimates of distribution shape

**Figure 4:**
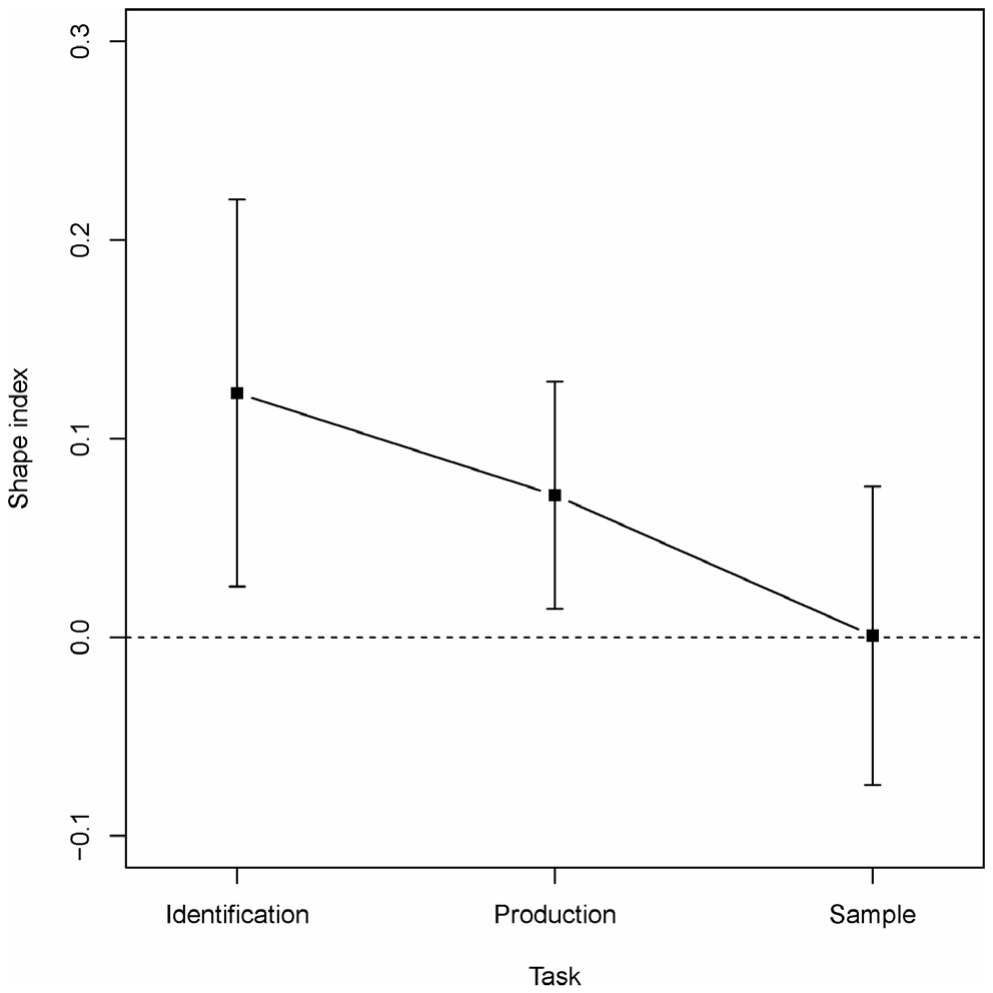
Performance in Experiment 1 in the three tasks given by the Shape Index. Dashed line indicates unbiased (uniform) estimates. Vertical bars denote 95%-confidence intervals.

The influence of presentation order and task format on accuracy was investigated by entering SI into a 3×3 mixed ANOVA with condition (U-B/B-U/UN) as between-subjects independent variable and task (production/identification/sample) as within-subjects independent variable. The significant main effect of task (*F*(2,90) = 5.11, *p* = .008), illustrated in Figure 4, indicated higher SI with the identification task (*M* = .12, *SD* = .34) than with the production (*M* = .07, *SD* = .2) and the sample (*M* = .01, *SD* = 2.59) tasks. A Scheffé's post hoc test showed that only the difference between performance in the identification and sample task reached significance. Neither the main effect of condition nor the interaction reached significance (*F*s<1). Figure 4 reveals a bias towards unimodality both in the identification and production tasks while there is no such bias in the sample task. Single sample *t*-tests showed that this bias was statistically significant (deviation from zero) in both these conditions (*p*<.05).

#### Sampling from Memory

Even though the instructions to the sampling task asked participants to disregard the recognition of specific values it is possible that they none the less used this information, when possible, to solve the task. The accurate performance in the sampling task may thus be due to specific memory of the values in *E* rather than by knowledge of the properties of *E*. To investigate this possibility a SI was calculated for the new (*T*) and old (*E*) values separately and entered into a 3×2 mixed ANOVA with condition (U-B/B-U/UN) as between-subjects independent variable and matrix type (new/old) as within-subjects independent variable. The analysis showed that neither the main effect of matrix type (*F*(1,45) = 1.61, *p* = .21; Old: *M* = .02, *SD* = .27; New: *M* = −.01, *SD* = .27) nor the main effect of condition or the interaction effect (both *F*s<1) reached significance (with the current design have a power of .99 of detecting a medium effect size for the new/old within-subjects main effect). This indicates that performance in the sample task was not related to the specific values seen during exposure.

### Discussion

In Experiment 1 we investigated if common memory effects influenced statistical judgments. We found no effect of the order in which the data was presented in any of the three tasks. This evidence seems to indicate that participants have access to all of the data at the time of a judgment and that the samples used to estimate statistical properties are not drawn conditionally on when the data is presented. A caveat to this conclusion is that our participants might not have been responsive to the underlying distribution at all but rather gave uniform estimates as the result of some bias or default strategy under ignorance. In Experiment 2 we addressed this possibility.

The results of Experiment 1 replicated previous results [1] with better performance in the production than in the identification task. Those results were further extended by showing that a task format (the sampling task) that is analogous to the suggested cognitive process (sampling from memory), will allow participants to perform at a higher level. It might be argued that the better performance in the sampling tasks is the result of a specific memory of the values shown in the exposure phase. However, the lack of old-new differences makes this interpretation less probable.

In both the identification task and the production task there was a tendency for participants' judgments to be biased towards unimodality. Previous research [1], [5] has argued that this is a consequence of a lazy cognitive process. However, the results might also arise if participants enter the task with a prior assumption that the data will be unimodally distributed. A second objective of Experiment 2 was to distinguish between these two possibilities. The results for the estimates of central tendency and variability indicated no differences between the presentation orders. However, while estimates of central tendency were fairly accurate (although slightly overestimated) variability was systematically and strongly underestimated. This is consistent both with previous findings [1], [8] and with what could be expected from a lazy cognitive algorithm.

Finally, as evident from the area of contingency judgments, having participants make judgments half-way through the observation phase sometimes has profound effects on primacy/recency shown in subsequent judgments. In Experiment 2 we were able to investigate if similar effects would appear for judgments of descriptive properties.

## Experiment 2

Experiment 1 indicated no traces whatsoever of memory effects on statistical judgments. The lack of effect might, however, be the result of insensitivity rather than responsivity to the underlying distribution. In Experiment 2 we aimed at distinguishing between these possibilities by interrupting the exposure phase with a test (the production test) to investigate if participants update their knowledge of the distributional properties of the experienced data. In addition to answering this question the design of Experiment 2 allowed us to evaluate the degree to which participants enter the task with any expectation of the distributional properties of the presented stimuli. Experiment 2 enabled us to examine whether or not such effects would be elicited by repeated judgments. It may, for example, be the case that people use their initial judgments as an anchoring hypothesis about the descriptive statistic, which is then not sufficiently adjusted by later information, resulting in a primacy effect. The results of an experiment with judgments of descriptive statistics may turn out in different ways, all with distinct interpretations; A) Initial and final judgments turn out similar, with uniform ratings in all conditions. This would show that the participants are in fact poor naïve statisticians, and that the overall uniform pattern is an effect of a bias in presence of ignorance. B) Initial judgments may correspond well to initial data, whereas final judgments converge slightly, but are biased towards the initial judgments. This would show an anchoring effect of making initial judgments, but sensitivity to data. C) Initial judgments may be separated, but fully converge on uniformity in the final judgments. This pattern of data would indicate a high performance level in the estimates. Participants would be both sensitive to data and able to update initial beliefs in view of changing information.

### Method

#### Participants

Participants were 42 undergraduate (15 male) students from Uppsala University (*M* = 23.1 years, *SD* = 6.7) receiving a movie voucher or course credits for participating.

#### Materials and procedure

Experiment 2 adopted the same design, materials, and procedure as Experiment 1 with one minor change. In Experiment 2 the exposure phase was interrupted after 60 values with a test (production task) after which participants experienced the remaining 60 values. Prior to the final test, after 120 presented values, participants were explicitly instructed to base their judgments on all of the 120 experienced values. Participants were randomly assigned to one of the three conditions with an equal number of participants (*n* = 14) in each condition.

### Results

#### Intermediate vs. Final test

**Figure 5:**
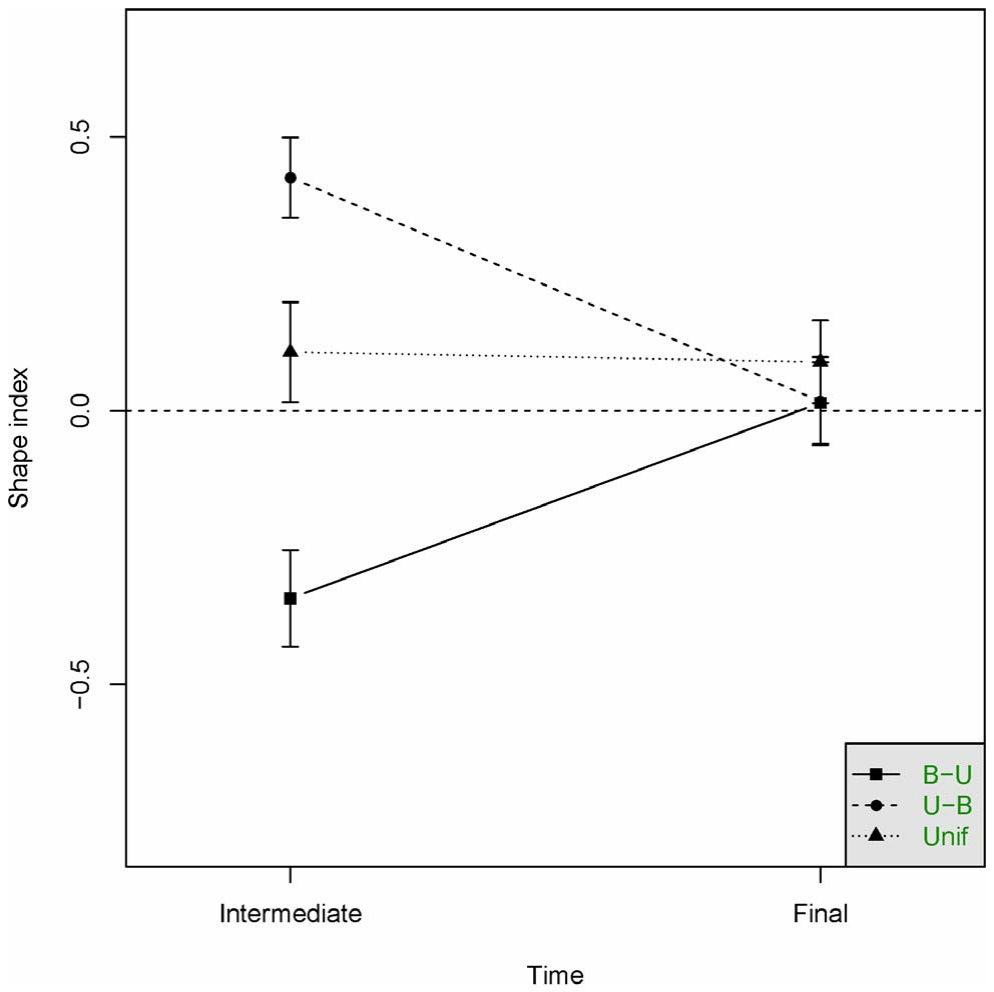
Performance in Experiment 2 in the Intermediate and Final test (production task) in the three conditions. Dashed line indicates unbiased (uniform) estimates. Vertical bars denote 95%-confidence intervals.

As in Experiment 1, SI was used as performance measure for knowledge of distribution shape. We compared participants performance in the production task in the intermediate and final test by entering SI as dependent variable into a 3×2 mixed ANOVA with condition (U-B/B-U/UN) as independent between-subjects variable and test time (intermediate/final) as independent within-subjects variable. The result, illustrated in Figure 5, indicated a significant main effect of condition (*F*(2,39) = 43.5, *p*<.001) and a significant condition by test time interaction (*F*(2,39) = 69.3, *p*<.001). As is evident from the figure, the interaction is due to participants in all three conditions reproducing a distribution consistent with the experienced data, both at the intermediate and final test. The main effect of test time was not significant (*F*<1).

#### Central tendency and variability

**Figure 6:**
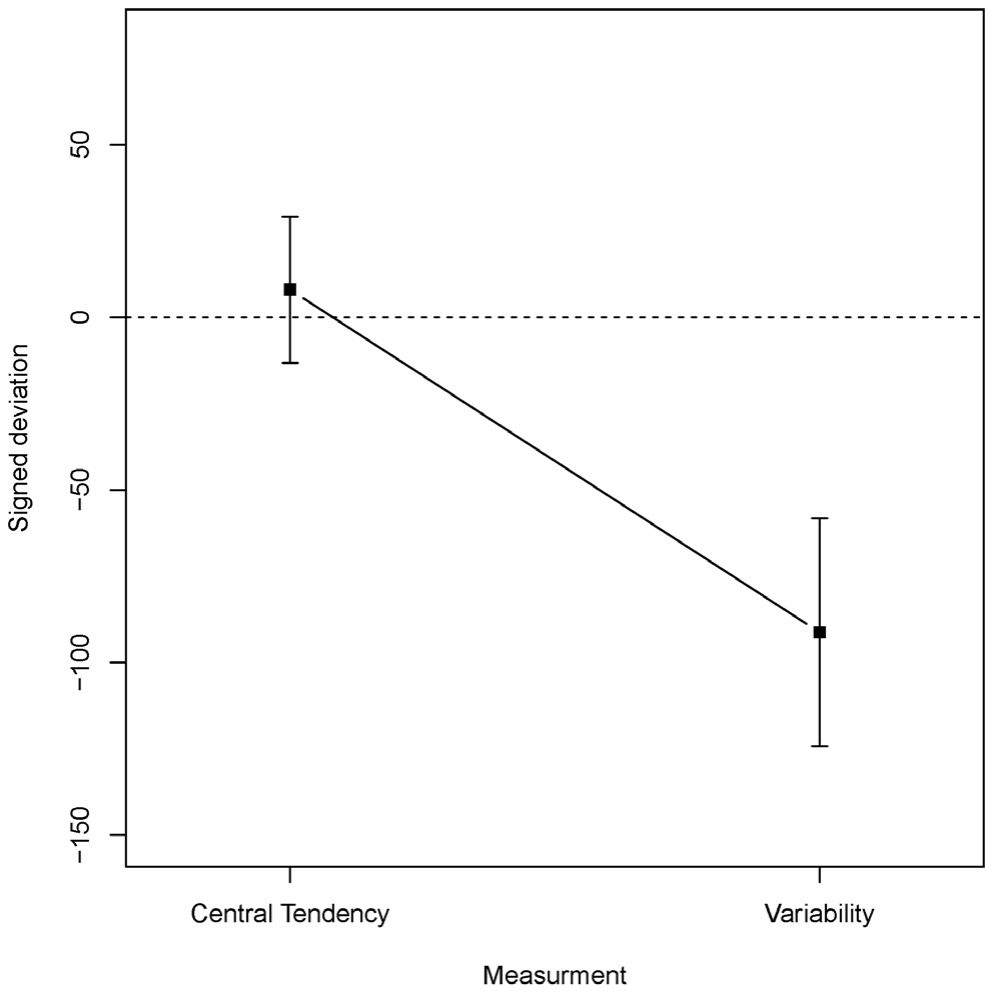
Signed deviation of estimates of Central tendency and Variability in Experiment 2. Dashed line indicates unbiased estimates. Vertical bars denote 95%-confidence intervals.

The accuracy and possible biases of estimates of central tendency and variability were investigated by entering the absolute and signed deviations respectively into two 3×2 mixed ANOVAs with condition (U-B/B-U/UN) as between-subjects independent variable and measurement type (central tendency/variability) as within-subjects independent variable. For the absolute deviation only the main effect of measurement type reached significance (*F*(1,37) = 21.0, *p*<.001, both other *F*s<1) with more accurate estimates of central tendency (*M* = 43.8, *SD* = 50.0) than of variability (*M* = 109.5, *SD* = 83.1). With regards to a bias the analysis revealed a significant effect of task (*F*(1,37) = 23.7, *p*<.001, both other *F*s<1). As is evident from Figure 6 there is no bias for estimates of central tendency while variability is underestimated.

#### Estimates of distribution shape

Performance in the final test over the three tasks was compared by performing the same analysis as in Experiment 1. The analysis showed a significant main effect of condition, U-B/B-U/UN, (*F*(2,39) = 4.0, *p* = .027) with a unimodality bias in the UN (*M* = .12, *SD* = .23) condition, but only a slight such tendency in the B-U (*M* = .02, *SD* = .2) condition, and a negative SI in the U-B (*M* = −.05, *SD* = .23) condition. A Sheffé post hoc test indicated that the UN/U-B difference was the only pairwise difference that reached significance. Neither the main effect of task (Identification: *M* = .03, *SD* = .3; Production: *M* = .04, *SD* = .14; Sample: *M* = .03, *SD* = .22) nor the interaction was significant (both *p*s>.32). Single sample *t*-tests revealed that only the unimodality bias in the uniform condition was statistically different from zero (*p*<.05).

#### Sampling from memory

As in Experiment 1 we compared old versus new values in the sampling task. A SI was calculated for the new (*T*) and old (*E*) values separately and entered into a 3×2 mixed ANOVA with condition (U-B/B-U/UN) as between-subjects independent variable and matrix type (new/old) as within-subjects independent variable. The analysis showed no significant old/new effect (*F*(1,39) = 0.05, *p* = .83; Old: *M* = .025, *SD* = .23; New: *M* = .03, *SD* = .22) and no interaction (*F*<1) indicating that performance in the sample task was not related to the specific values seen during exposure. There was, however, a main effect of condition (*F*(2,39) = 3.59, *p* = .04) with a more positive deviation from the normative distribution in the UN (*M* = .14, *SD* = .27) than in the B-U (*M* = −.01, *SD* = .13) and U-B (*M* = −.05, *SD* = .21) conditions.

#### Individual Differences

For all individuals in both experiments we obtained measures of various potentially interesting cognitive abilities. We measured general cognitive ability (Raven's matrices), graph literacy [46], long-term memory for numbers (free recall), and working memory (digit span). Because no effects of the experimentally manipulated independent variables were found in either study, we collapsed all data from both studies in order to increase power when undertaking an individual differences analysis. We entered the four measures of abilities above as predictors in multiple regression analyses with the aim to predict dependent variables of the experiments. Generally, the regression weights were low and non-significant. However, we found two interesting correlations. With the SI measure obtained from the graph task as dependent variable, graph literacy was a significant predictor (*β* = −.23, *t*(79) = 2.05, *p*<.05). This indicates that a bias towards unimodality obtained with this measure is larger for participants who perform less well in interpreting graphs. With the difference between old and new stimuli in the sample task (of the mean absolute error between the normative and chosen stimuli) as dependent variable long-term memory for numbers was a significant predictor (*β* = .27, *t*(79) = 2.4, *p*<.05.). This suggests that participants with good memory for numbers can capitalize on this ability and perform better for previously presented stimuli than for new stimuli in the sample task.

### Discussion

Experiment 2 investigated two questions. First, Experiment 1 indicated little influence of memorial effects but could not rule out that this was the result of insensitivity to the underlying distribution. Experiment 2 explored this possibility by introducing an intermediate test. The results, illustrated in Figure 5, suggest both that our participants are sensitive to the underlying distribution and that they are efficient in incorporating new data as it is presented. It is possible that the intermediate test makes the participants more aware of the purpose of the data presentation and that they thereby will produce a more accurate representation of the underlying distribution. However, performance on the final test was very similar to Experiment 1, indicating only a small effect by the introduction of an intermediate test.

Second, Experiment 2 investigated if participants enter our task with expectations about the properties of the presented data. Previous research has indicated that such possible expectations are likely to be unimodal [47]. As is evident from the results of the intermediate test, illustrated in Figure 5, participants are clearly data-driven, but there is a bias towards unimodality in the uniform condition. However, in terms of prior expectations our participants seem to have very weak priors concerning the presented data.

In contrast to Experiment 1 we found no effect of task in Experiment 2. Previous research has suggested that being engaged in the production format prior to performing the identification task boosts performance in the latter by allowing for a more abstract representation being formed [1]. It is possible that the lack of effect is due to the introduction of the intermediate test. This would need to be explored in future research. There was, however, an effect of condition with participants in the uniform condition performing slightly worse than those in the other two conditions. This was due to the tendency for a unimodality bias in the uniform condition while no such bias was observed in the other two conditions.

Experiment 2 further replicated two of the findings from Experiment 1. First, while estimates of central tendency were fairly accurate and unbiased, estimates of variability were inaccurate and underestimated the normative variability. Second, there was no old-new difference in the sample task indicating that the observed results are the product of an inference process rather than specific memory of values seen during exposure.

## General Discussion

The results of the first experiment showed that our intuitive statisticians were remarkably resistant to memory mechanisms of either primacy or recency effects. Perhaps most remarkable is the lack of effects for variability ratings, since this variable undergoes a dramatic change from the first to the second half of the information sequence in the present design. The results of this experiment could, however, alternatively have been due to a response bias towards uniformity in absence of learning. Previous research [1], [5] has indicated a decent level of performance of participants under similar conditions, which makes this alternative interpretation less plausible. To rule out this entirely, however, in Experiment 2 we measured participants' meta-knowledge about the distributions half-way through the information sequence when the different experimental groups had observed extremely different conditions (i.e., either bimodal, uniform or unimodal). The results of these initial ratings showed that participants were far from ignorant, but diverged in their ratings in the direction and approximate magnitude suggested by the normative Shape Index associated with the experienced numeric information. As for the final judgments, again data showed that participants did not ”get stuck” in hypotheses guided by their initial observations, but rapidly converged towards the uniform normative distribution of the entire data set.

A lazy cognitive algorithm would be expected to perform well in a changing environment only when there is little temporal dependency in the presented data and no systematic memory effects. The results of both experiments indicated no systematic memory effects, which is consistent with the idea that people rely on memory processes analogous to the sampling from LTM in the NSM, in which the sample is modeled independent of temporal sequence. The lack of both order effects and effects of making explicit judgments during stimulus observation is in contrast with observations from the contingency learning paradigm, which suggest that other processes, possibly more complex or associative may be operating when one is trying to estimate whether it is possible to predict variable y from the changes in variable x. It would be interesting to further investigate the nature of these differences.

In the present study participants experienced 120 values on a trial-by-trial basis and were required to report their knowledge of the properties of these values shortly after. While we found no memory effects it is possible, however, that a larger set of values or a longer retention time might introduce such effects. It is an interesting venue for future research to investigate the limit at which this immunity to memory effects breaks down and thereby investigate the capacity of the lazy cognitive algorithm. Whenever observing a lack of effect, this may of course also depend on a lack of statistical power to reject the null hypothesis. As for the within-subjects tests reported above this power as reported above is high. For the main between subjects comparison the power is approximately .7 to reveal a large effect (RMSSE = .5). There may be a smaller effect that we fail to detect with the limited sample size we rely on. However, there are no trends in the data suggesting that our results are due to limited statistical power. In addition, we replicate this finding over two experiments. Nevertheless, it would be nice to replicate this finding in future research with higher power for the between-conditions comparison.

In the first experiment, the subjective shape index was slightly, but statistically significantly biased towards unimodality (i.e., positive). In the second experiment there was a unimodality bias only in the uniform condition. The reasons for this difference is unclear, but we have previously found the unimodality bias to be elusive and sensitive to changes in task characteristics [1], [5]. One possibility is that the initial frequency judgments may make participants more data driven when a more extreme (i.e., the bimodal-unimodal and unimodal-bimodal conditions) distribution precedes the judgment rather than when the judgment is made successive to a probably less psychologically “salient” uniform distribution. Although this explanation is clearly speculative, we have previously found [1] that having people make frequency judgments of extreme distributions may indeed enhance performance in subsequent statistical judgments. Although temporal sequence effects do not seem to affect the processes, there may well be other effects such as availability that will distort sampling from long-term memory. Future studies should investigate such effects. To sum up, we conclude from the results of the present study that there is no evidence for a dependency between temporal encoding time of data in long-term memory and subsequent judgments. This is in line with a “random sampling” metaphor of retrieval of exemplars for judgments by the naïve intuitive statistician. Overall, participants performed quite well and apparently updated their judgments in a seemingly rational manner.
